# Analysis of ten-year teaching evaluation of oral microbiology lab curriculum

**DOI:** 10.1186/s12909-024-05298-1

**Published:** 2024-03-19

**Authors:** Yu Xu, Xingji Ding, Wenhui Wang, Yazhuo Li, Min Nie

**Affiliations:** 1 School and Hospital of Stomatology, Wuhan University, Wuhan, Hubei China; 2 The State Key Laboratory Breeding Base of Basic Science of Stomatology, Hubei Province & Key Laboratory of Oral Biomedicine (Wuhan University), Ministry of Education, School and Hospital of Stomatology, Wuhan University, Luoyu Road 237, Wuhan, Hubei 430079 China

**Keywords:** Oral microbiology, Laboratory teaching, Cariogenic microorganisms

## Abstract

**Background:**

Based on the updated teaching philosophy of oral microbiology, Wuhan University School of Stomatology initiated a reform in the teaching of oral microbiology in 2009. As part of this reform, an oral microbiology laboratory course was introduced to cultivate students' fundamental skills, professional competence, comprehensive abilities, and innovation capabilities through experimental design. This paper provides thorough examination of the teaching experiment findings from 2013 to 2022, a ten-year timeframe, building on earlier data.

**Methods:**

The curriculum targets fourth-year undergraduate students in a five-year program and adopts a cooperative learning approach. The experimental teaching mainly involves four parts: plaque collection and processing, isolation and cultivation of dental plaque bacteria, staining and biochemical identification of dental plaque bacteria. This article presents a comprehensive analysis of the student experiment results from 2013 to 2022. Statistical analysis was conducted using the chi-square test to assess whether there were any differences in students' experimental grades between different years. A significance level of *P* < 0.05 was considered statistically significant. Additionally, we evaluated the impact of teaching methods and educational systems on improving students' practical skills and overall innovative abilities.

**Results:**

The performance of 664 undergraduate students showed improvement in the oral microbiology laboratory course, with a noticeable decrease in the proportion of "C" grades in Experiments 2, 3, and 4 compared to Experiment 1. These results indicate that the laboratory course enhanced students' academic achievements, aiding their understanding and mastery of course content, and received positive feedback from the students.

**Conclusion:**

This lab curriculum, through systematic laboratory teaching and practical experience, contributes to the enhancement of students' professional skills and research abilities. It fosters students' interest in scientific research and improves the quality of dental education.

## Introduction

Oral microbiology is one of the representative foundational disciplines in dentistry, it is an interdisciplinary subject that combines microbiology and stomatology, encompassing areas such as microbiology, caries, endodontics and periodontal diseases. It possesses the professional characteristics of microbiology and holds a close connection with oral clinic practice. In 1960, the School of Dentistry (now the School of Dental Medicine) at the University at Buffalo established the first department of Oral Biology in the United States. This department was dedicated to provide graduate biomedical research education and basic oral science education, making it the pioneering dental department in this field. Oral biology is a relatively new discipline that gradually emerged in some of the western developed countries during the early 1980s. Currently, oral biology courses are offered in dental colleges across most countries in North America and Europe [1, 2]. Oral microbiology is an important branch of oral biology. In 2007, the National Institutes of Health (NIH) officially launched the "Human Microbiome Project" to study the relationship between the human microbiome and disease and health. Although we now understand well how oral microbiology is closely linked to oral diseases, historically, education in oral microbiology began later compared to other specialties in oral medicine. Establishing a laboratory requires significant time, funding, and effort. Unfortunately, in many developing countries, the curriculum is still not available in many areas due to limited resources like, insufficient faculty, finite curricular hours, and financial constraints. In the 1980s, oral biology was first introduced in China, the largest developing nation in the world. Currently, the development is still imbalanced in more than 80 medical schools of stomatology in China although the teaching of oral microbiology is constantly rising. It is worth noting that some departments have not yet implemented the oral microbiology curriculum. In 2009, Wuhan University became one of the earliest universities in China to launch the oral microbiology lab curriculum. In 2012, the Chinese Journal of Microecology published the experimental teaching paper titled "Exploration of Oral Microbiology Experiment Teaching System," marking the first publication of its kind in China [3]. Our experiences can serve as a valuable reference for other dental schools seeking to establish similar courses.

Multiple studies have confirmed the crucial role of oral microorganisms in the onset and progression of various oral infectious diseases and systemic diseases [4–6]. An imbalance in oral microecology can trigger a range of oral diseases, including caries, periapical inflammation, periodontal disease, and oral cancer. Furthermore, it is strongly associated with systemic diseases such as diabetes, rheumatoid arthritis, and cardiovascular disease [7]. The oral microbial community represents a typical biofilm, and among the biofilm-induced diseases, dental caries and periodontal disease have been extensively studied [6]. The 4th National Oral Health Survey revealed that the occurrence of dental caries ranged from 50.8% to 98.0% across different age groups [8]. The global burden of dental caries remains substantial [9, 10]. Consequently, we established the laboratory curriculum system centered around caries microorganisms.

Our educational philosophy is "to train people who can utilize biological thinking and methods (techniques) to address the problems significant to dentistry ". We aim to cultivate students' awareness that all medical research originates from clinical settings and serves the purpose of enhancing clinical practice. As a crucial mandatory course for undergraduate students, the practical component of our curriculum enables students to apply theoretical knowledge through hands-on experimentation. Aligned with our updated educational concept and the evolving needs of the medical field, more emphasis is placed on fostering students' capabilities [11]. We are also increasing the number of undergraduates with authentic early research experience, nurturing their enthusiasm for science and research, and laying a foundation for medical undergraduates' future scientific research [12, 13].

In our previous study, we analyzed data from 2013 to 2022 and discovered that the oral microbiology lab curriculum helped enhance students' understanding of oral biology theory, as well as developing their hands-on skills and scientific research thinking [14]. There are also studies indicating that simple and short microbiology practical can enhance students' understanding of microbiology [15]. Building upon these findings, we have conducted further observations and evaluations of the experimental course from 2013 to 2022. This long-term observation aims to validate our hypothesis and provide robust evidence for the effectiveness of the curriculum. Furthermore, we have implemented a new experimental education system that includes a component on experimental design. We also encourage students to participate in innovative training programs to foster their scientific research thinking and their ability to identify and address specific clinical problems using their acquired knowledge. Currently, this concept is continuously evolving and being refined.

## Hypothesis

The oral microbiology laboratory curriculum will help: (1) enhance students' mastery of professional knowledge, (2) improve students' practical skills, (3) strengthen students' innovation and comprehensive abilities, and (4) enhance the quality of dental education.

## Methods

### Participants and data collection

The performance data of 664 students in oral microbiology lab sessions from 2013 to 2022 were retrospectively evaluated in our study. The number of students in each year was as follows: 2013 (*n* = 56), 2014 (*n* = 67), 2015 (*n* = 53), 2016 (*n* = 52), 2017 (*n* = 53), 2018 (*n* = 68), 2019 (*n* = 78), 2020 (*n* = 77), 2021 (*n* = 79), and 2022 (*n* = 81). Students in the fourth year of a five-year bachelor's program are the target audience for the curriculum.

This entire study was submitted to and reviewed by the Medical Ethics Committee of Wuhan University, which subsequently waived the need for ethical approval. All 664 participants were informed of the aims of the evaluation and informed consent in the research.

### Teaching system

**Fig. 1:**
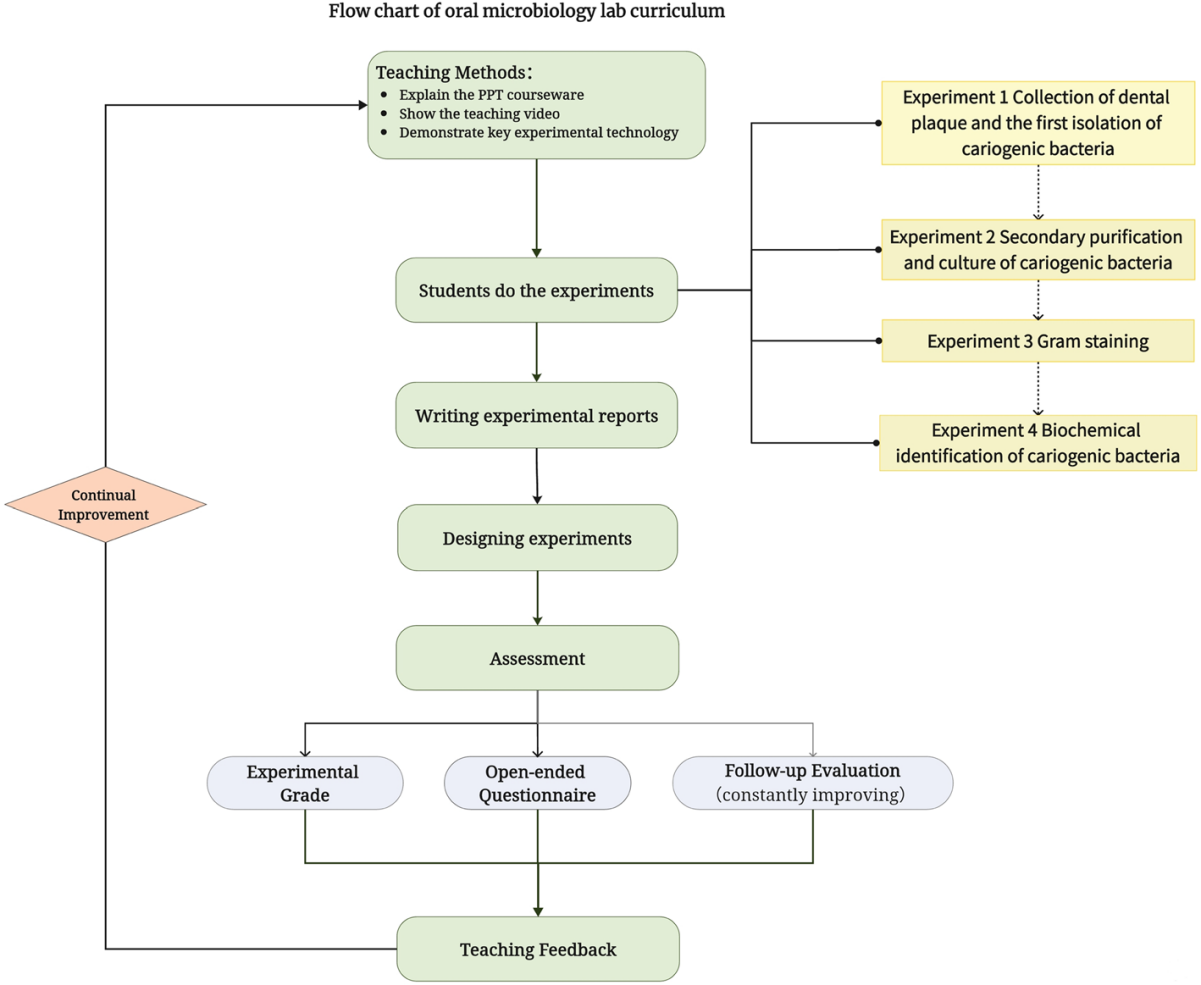
Flow chart of oral microbiology lab curriculum

The new teaching approach adheres to the same four teaching modules as before: basic, professional, comprehensive, and innovative experimentation. Nevertheless, as a result of the absence of prior courses focused on fostering inventive aptitude, we endeavor to incorporate a novel component—the inclusion of experimental design—to cultivate students' innovative thinking. The cumulative duration of the instructional period for the experiments amounts to 15 h. The flow chart describes our curriculum design (Fig. 1).

The teaching system utilizes a three-dimensional teaching structure and establishes a model consisting of "experimental operation unit – small experiment – experimental technique system – systematic experiment – design experiment ". The experimental operation unit serves as the fundamental component of the experiment, encompassing tasks such as sample collection, slide preparation, staining, microscopic analysis, sterilization, dilution, inoculation, culture. Mastery of the experimental operation unit is crucial for the successful implementation of oral microbiology experiments. A thorough understanding and proficiency in the experimental operation unit is a necessity for conducting subsequent experiments. The small experiment consists of experimental operation units, such as the Gram staining experiment, which includes experimental units such as smear fixation, dyeing, washing, drying, microscopic examination. The experimental technique system comprised of a collection of small experiments, such as microbial pure culture technology, microscopic technology, aseptic operation technology, and more. Systematic experiment involves the utilization of an experimental technique system, necessitating students to acquire proficiency in a range of experimental operation approaches. The tool addresses a particular scientific issue, such as the microbial biochemical identification experiment in this training module, which demands a greater level of proficiency in experimental knowledge from students. Lastly, the design experiment represents the pinnacle of the teaching model. It challenges students to apply their accumulated knowledge and skills to design experiments that aim to address specific research questions or solve clinical problems. We instructed the students to voluntarily assemble into teams consisting of 3–4 individuals and provide an experimental design report pertaining to the correlation between oral bacteria and oral illnesses subsequent to the class. The outcomes of the four experiments are evaluated by the identical instructor, and the remarks on the formulated experiment report are excluded from the ultimate score. A platform for open communication is established for teachers to enhance the curriculum based on students' achievements and the messages received.

The teaching method employed in the curriculum begins with teachers providing explanations using slides to introduce the relevant concepts and theories, followed by students viewing the instructional video. Subsequently, the teacher displays the key technology, and finally the students conduct the experiment and submit the experiment report.

### Teaching contents

The contents of experimental teaching include: Sampling and processing of dental plaque, isolation and cultivation of cariogenic bacteria, staining and biochemical identification of cariogenic bacteria.

#### Experiment 1 Collection of dental plaque and the first isolation of cariogenic bacteria

The first step to isolate cariogenic bacteria and identify *Streptococcus mutans* is to collect dental plaque, which depends on students' clinical examination skills [16]. The training utilizes a paired paradigm where two students exchange clinical samples with each other. The students rinse their mouth with clean water to remove food residues, and then use a probe or sterilized toothpick for plaque sampling. Samples were collected and vortex oscillation and ultrasonic oscillation were used successively to disperse plaque or bacterial clumps. Then dispersing the specimens 10 times in series, ensuring strict adherence to aseptic techniques throughout the process. Streak inoculating on Mitis salivarius-bacitracin (MSB) agar selective petri dish and incubate under anaerobic condition at 85% N_2_, 15% CO_2_ and 37 ℃ for 48h. Finally, observe the bacterial colonies on the primary culture plate, distinguish the cariogenic bacteria from the non-cariogenic bacteria under the anatomical microscope, and record the morphological characteristics of the colonies, including shape, size, thickness, edge, color, transparency, etc. Scoring based on the outcome of Experiment 1 (Table 1).

**Table 1:** Rating criteria of Experiment 1

Grade	Criterion	Capability evaluation
A	culture cariogenic bacteria successfully	basic ability + professional ability + comprehensive ability + innovation ability are good
B	culture other bacteria	professional ability is inadequate
C	culture no bacteria	basic ability + professional ability are inadequate

#### Experiment 2 Secondary purification and culture of cariogenic bacteria

Selecting typical colonies of *S. mutans* from the original culture plate, transferring it to BHI liquid medium for enrichment under aseptic operation and anaerobic culture for 24h. Supplement with bacterial sources for Students who scored "C" in the previous experiment. Scoring based on the outcome of Experiment 2 (Table 2).

**Table 2:** Rating criteria of Experiment 2

Grade	Criterion	Capability evaluation
A	Two copies success	Basic ability is good
B	One copies success	Basic ability is inadequate
C	All failure	Basic ability is poor

#### Experiment 3 Gram staining

Take the culture solution of purified cariogenic bacteria for Gram staining. Scoring based on the outcome of Experiment 3 (Table 3).

**Table 3:** Rating criteria of Experiment 3

Grade	Criterion	Capability evaluation
A	identify successfully	Basic ability is good
C	identify unsuccessfully	Basic ability is poor

#### Experiment 4 Biochemical identification of cariogenic bacteria

**Fig. 2:**
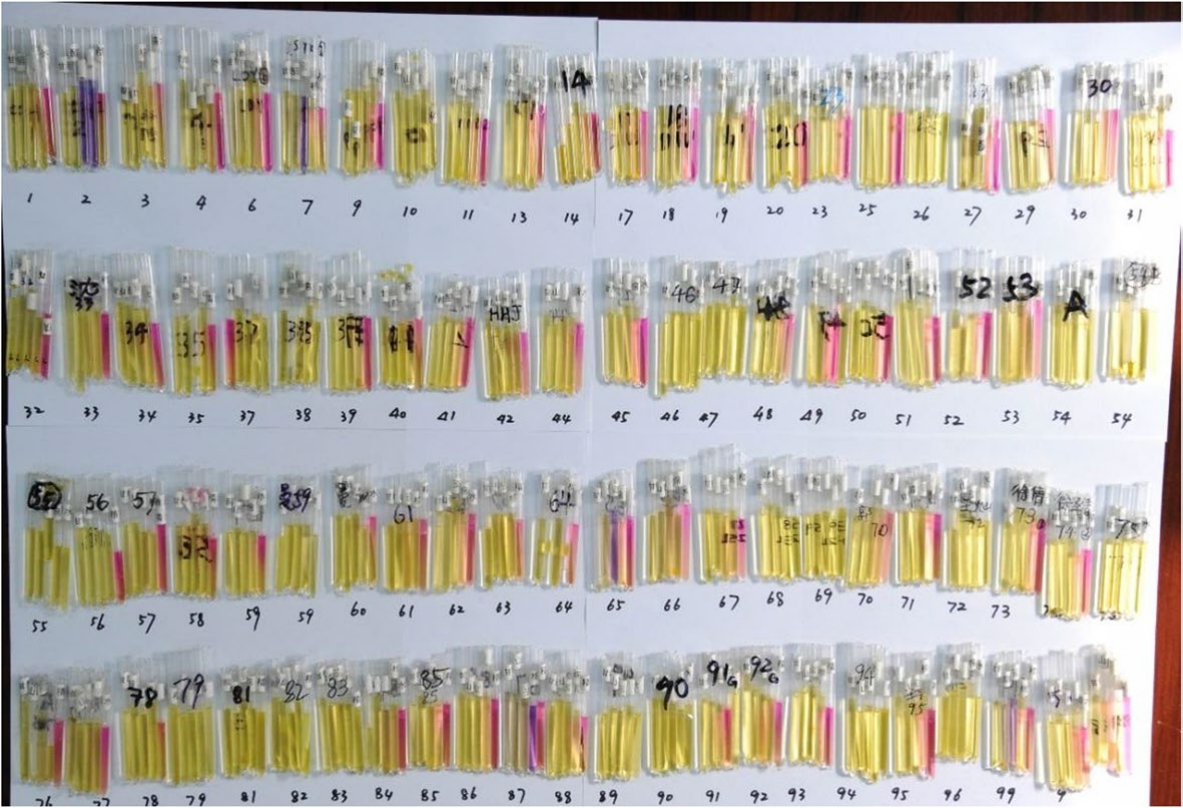
Partial results of biochemical identification of cariogenic bacteria. *The figure demonstrates the biochemical identification results of some students' Streptococcus mutans. From left to right, fermentation tests were performed using mannitol, sorbitol, raffinose, melibiose, and arginine sugar tubes. To obtain an A rating, either five yellow sugar tubes or four yellow sugar tubes and one red sugar tube are required

Put 10μL of bacterial liquid cultured in liquid medium for biochemical identification, add mannitol, sorbitol, raffinose, melibiose and arginine sugar tubes respectively, and anaerobic incubation for 24h. The first four sugar tubes turn from purple to yellow when positive, indicating fermentation, while no color change indicates a negative result. The arginine sugar tube turns from red to yellow when negative, indicating no fermentation, while no color change indicates a positive result. Observe and analyze the experimental results, summarize the reasons for success and failure. Scoring according to the result of Experiment 4 (Table 4). The results are shown in Fig. 2. Scoring based on the outcome of Experiment 4 (Table 4).

**Table 4:** Rating criteria of Experiment 4

Grade	Criterion	Capability evaluation
A	All success	basic ability + professional ability + comprehensive ability + innovation ability are good
B	Partial success	professional ability is inadequate
C	All failure	basic ability + professional ability are inadequate

### Statistical analysis

Data were analyzed using SPSS Version 24 (IBM Corp, Armonk, NY). Chi-square test was used to analyze whether there was any difference in scoring rate among experimental classes from 2013 to 2022. *P* < 0.05 was considered statistically significant.

## Results

### Part one experimental teaching achievements of oral microbiology

**Fig. 3:**
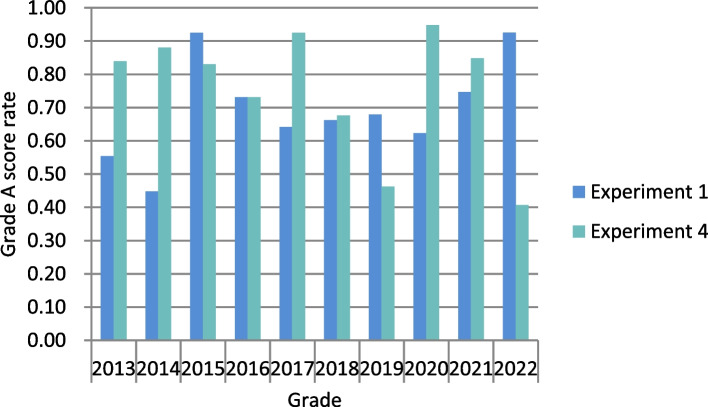
Experiment 1 and Experiment 4 Grade A Score Rate

Statistics of the teaching results of oral microbiology lab curriculum for 664 undergraduates majoring in stomatology in Wuhan University from 2013 to 2022, assessment are summarized in Table 5. Table 5 shows the score proportion of all students in the four experiments over the past decade, providing an overall dataset. From the table, we can observe a high proportion of A grades in Experiment 2 and Experiment 3 (93.5% and 99.2%, respectively). In Experiment 3, which involves gram staining, only A and C scores are given as it is divided into successful and unsuccessful categories. See Table 6 for the situation year by year from 2013 to 2022. See Fig. 3 for the variation of scoring rate of A in Experiment 1 and Experiment 4.

**Table 5:**
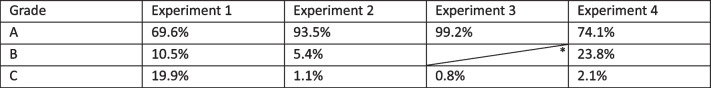
Overall assessment of oral microbiology experiments

*The results of Experiment 3 are categorized as successful (**A**) or unsuccessful (**C**), with no **B** grade assigned

**Table 6:** Results of Oral Microbiology Experiment Courses from 2013 to 2022

	Score	2013	2014	2015	2016	2017	2018	2019	2020	2021	2022
Experiment 1	A	31(55.4%)	30(44.8%)	49(92.5%)	38(73.1%)	34(64.2%)	45(66.2%)	53(67.9%)	48(62.3%)	59(74.7%)	75(92.6%)
	B	6(10.7%)	7(10.4%)	4(7.5%)	6(11.5%)	5(9.4%)	15(22.1%)	2(2.6%)	12(15.6%)	12(15.2%)	1(1.2%)
	C	19(33.9%)	30(44.8%)	0(0.0%)	8(15.4%)	14(26.4%)	8(11.8%)	23(29.5%)	17(22.1%)	8(10.1%)	5(6.2%)
Experiment 2	A	47(83.9%)	66(98.5%)	40(75.5%)	52(100.0%)	53(100.0%)	64(94.1%)	77(98.7%)	62(80.5%)	79(100.0%)	81(100.0%)
	B	9(16.1%)	1(1.5%)	7(13.2%)	0(0.0%)	0(0.0%)	3(4.4%)	1(1.3%)	15(19.5%)	0(0.0%)	0(0.0%)
	C	0(0.0%)	0(0.0%)	6(11.3%)	0(0.0%)	0(0.0%)	1(1.5%)	0(0.0%)	0(0.0%)	0(0.0%)	0(0.0%)
Experiment 3	A	55(98.2%)	67(100.0%)	53(100%)	52(100.0%)	53(100%)	65(95.6%)	78(100.0%)	77(100%)	78(98.7%)	81(100.0%)
	C	1(1.2%)	0(0.0%)	0(0.0%)	0(0.0%)	0(0.0%)	3(4.4%)	0(0.0%)	0(100%)	1(1.3%)	0(0.0%)
Experiment 4	A	47(83.9%)	59(88.1%)	44(83.0%)	38(73.1%)	49(92.5%)	46(67.6%)	36(46.2%)	73(94.8%)	67(84.8%)	33(40.7%)
	B	5(8.9%)	2(2.9%)	5(9.4%)	14(26.9%)	4(7.5%)	22(32.4%)	42(53.8%)	4(5.2%)	12(15.2%)	48(59.3%)
	C	4(7.1%)	6(9.0%)	4(7.5%)	0(0.0%)	0(0.0%)	0(0.0%)	0(0.0%)	0(0.0%)	0(0.0%)	0(0.0%)
Total		56	67	53	52	53	68	78	77	79	81

The statistical analysis of Experiment 1, conducted from 2013 to 2022, revealed a significant difference in the scoring rate of A among different years (χ2 = 62.580, *P* < 0.05). Similarly, when examining the results of Experiment 4, which focused on the A score, a significant difference was observed in the score rate among different years (χ2 = 123.243, *P* < 0.05). For the four different experiments, there are significant difference in the rate of grade A scores (χ2 = 312.68, *P* < 0.05); experiments 2 and 3 have higher rates of grade A scores compared to experiments 1 and 4, indicating that students' grades fluctuate based on the difficulty level of the experiments. Simultaneously, there are also significant difference in the rate of grade C scores for the four different experiments (χ2 = 308.289, *P* < 0.05). Grade A represents the most outstanding group of students, while grade C represents those with lower basic abilities, serving as the lower limit of teaching outcomes. Experiment 1 has the highest rate of grade C scores, and Experiment 4 is the most challenging. However, after training in the first three experiments, the rate of grade C scores for Experiment 4 decreases, indicating an improvement in students' basic experimental skills.

### Part two student self-assessment and teaching feedback results

We offer a post-class open communication platform. By examining students' understanding of the experimental course on oral microbiology, it has been determined that students have given positive feedback for the course. Within the experiment report, students conducted self-evaluations and expressed their personal perspectives regarding the course:

"This bacterial isolation and identification experiment can offer insights for our forthcoming experiments involving bacteria";

"When we gather plaque to cultivate bacteria that cause tooth decay, we recollect the favorable locations for cavities that we learned about in the theoretical course, and subsequently choose the sites of these cavities and gaps ";

"The experimental course emphasized the importance of aseptic operation in order to prevent contamination. This included being cautious when opening the lid of the petri dish to avoid excessive exposure to bacteria, ensuring proper sterilization of the inoculation ring, and conducting all operations on the aseptic operation table ";

"While some instruments and basic operations may seem straightforward, it is crucial to adhere strictly to the specified requirements and develop good experimental habits. Adhering to proper experimental practices is crucial for ensuring the success of an experiment ";

A group of students creatively linked the culture of Streptococcus salivarius with the selective culture of dental plaque in order to distinguish between caries-causing bacteria and non-caries-causing bacteria. Alternatively, they explored the possibility of manipulating the oral microbiome through dietary modifications to enhance oral health. Simultaneously, students aspire to augment their access to experimental apparatus and eagerly anticipate engaging in laboratory work for the purpose of conducting in-depth investigations and acquiring knowledge under the guidance of their lecturers.

## Discussion

Modern concept holds that the occurrence and development of dental caries is related to the imbalance of oral plaque biofilm microecology. Changes in the plaque microenvironment lead to alterations in the colony structure. *Streptococcus mutans*, one of the major etiological agents of human dental caries, primarily resides in the biofilm on the tooth surface [17–20]. Its simplicity in gathering data renders it one of the fundamental components of our experiment.

Building upon previous basic, professional, comprehensive and innovative experiments, we have implemented additional enhancements and developed a three-dimensional teaching system called the "experimental operation unit – small experiment – experimental technique system – systematic experiment – design experiment" teaching system. The four experiments progress sequentially and are interconnected. The preceding experiment serves as the basis for the subsequent experiment, not only raising the level of complexity but also intensifying the level of challenge and fascination in scientific investigation.

Experiment 1 belongs to the “experimental technique system”, which encompasses microscope technology, aseptic operation technology, and fundamental experimental units such as sampling, dilution, and inoculation. This experiment demands students to possess advanced basic experimental skills. Conducting oral examinations and collecting samples of dental plaque help students to develop their professional abilities, while isolating bacteria that cause tooth decay assesses their proficiency in conducting fundamental microbiological experiments. The success of the experiment is determined by factors such as the presence and sterility of the sample, the technique used to draw the lines, and the dexterity of the operation. Simultaneously, documenting the form, size, thickness and edge of colonies with professional terminology in scientific research records also fosters students' objective and rigorous scientific research. The statistical analysis reveals variations in the scoring rate of A throughout different years. Based on the data analysis presented in Table 6, the grade A score rate for Experiment 1 was at its lowest in the decade during 2013 and 2014. However, notable improvement has been observed from 2015 onwards. There was no significant difference between the scores from 2016 to 2021 (χ2 = 3.84, *P* = 0.573), indicating a stable performance among students during these years. This observation may suggest that the increase in teachers' experience and the adoption of effective teaching methods have had a positive impact. The improvement in scores in 2015 and 2022 could be attributed to chance.

Experiment 2 once again emphasized the importance of aseptic techniques, further enhancing students' fundamental experimental skills and fostering scientific research habits. We provided additional bacterial sources to the students who did not succeed in the first experiment, resulting in a success rate of over 90%.

Experiment 3 serves as a “small experiment” within the three-dimensional experimental structure system, focusing on strengthening students' fundamental microbiology experimental skills. The level of difficulty in this experiment is relatively low. After the training received during Experiment 1 and Experiment 2, students' proficiency and operational competence have improved. As a result, the success rate for Experiment 3 reaches an impressive 99.2%. Experiment 2 and Experiment 3 are fundamental microbiology experiments known for their ease of access and the possibility of achieving high scores. This highlights the fact that microbiology education aids in consolidating basic experimental skills and fostering students' self-assurance in their learning abilities.

Experiment 4 is a “systematic experiment” aimed at identifying cariogenic microorganisms, which poses a challenging aspect of experimental instruction. Table 6 displays variations in the ratio of the score of A in Experiment 4. The pupils' academic performance in 2019 (46.2%) and 2022 (40.7%) is significantly inferior compared to other years. Experiment 4 is the most challenging and extensive experiment, demanding a high level of students' overall ability. The students' grades tend to be unpredictable throughout this period. Therefore, it should be regarded as one of the key points of oral microbiology curricular rejuvenation. Endeavors must be undertaken to establish a uniform teaching approach, enhance the integration of theoretical information, and standardize the presentation of experimental procedures. Simultaneously, students are encouraged to promptly compose experimental reports, contemplate and summarize both the achievements and shortcomings of the experiment.

The experimental results demonstrate that the four selected experiments are characterized by continuity, comprehensiveness, and representativeness, hence facilitating students' profound comprehension of cariogenic bacteria in plaque biofilm. As part of their homework, students are required to write experiment reports after each session. Following this series of training, we instructed the students to form groups, to search relevant literature, propose themes related to oral microbiology and disorders that piqued their interest, and subsequently develop an experimental report. Experimental design represents the pinnacle of experimental methodology, as it involves tackling specific scientific problems and considering various factors comprehensively. For instance, certain student groups have devised experiments such as "The relationship between recurrent Aphthous ulcer and oral microbiome" and "The influence of Smoking on oral microbiome of healthy people". These projects require students to write experimental reports in the form of scientific papers, necessitating competent laboratory skills and the ability to independently consult, read, and comprehend scientific literature. This can facilitate the acquisition and use of scientific methodologies by undergraduate students, enhancing their capacity for critical thinking, while also fostering their aptitude for collaboration and effective communication, all of which are crucial in the realm of scientific inquiry [13]. Undertaking such challenges can be daunting for medical undergraduates. To mitigate the significant strain on students, the final grade does not incorporate the outcome, which encourages students to be bold and innovative to a certain extent.

Recent suggestions on biology education have highlighted the significance of providing authentic research opportunities at the beginning of undergraduate studies to enhance the quantity and caliber of biology students. Participating in research experiences can enhance students' enthusiasm for scientific inquiry, improve the retention rate of biology majors, enable students to effectively understand and communicate science, develop critical thinking skills, and better prepare the next generation of scientists while fostering persistence in scientific pursuits [13, 21–24]. A survey conducted at CEU Cardenal Herrera University revealed that the inclusion of authentic research experiences in microbiology courses had a positive impact on students. The results indicated improvements in critical thinking skills, knowledge of research design, and a deeper understanding of microbial analysis methods [25]. Our educational philosophy aligns with this approach, we strongly encourage students with a keen interest in research to approach their tutors and seek opportunities to participate in laboratory work. Considering the limitations of laboratory resources, it may not be feasible for every student to carry out their experimental designs. A group of students presented their experimental proposals to their tutors, which were acknowledged and granted permission to use the laboratory for conducting experiments. Furthermore, another pathway for undergraduate students to enter the laboratory is by applying for the Ministry of Education's College Students' Innovative Entrepreneurial Training Plan Program to gain the opportunity for research training under the guidance of tutors. The school considers it crucial to expedite the integration of undergraduates into the professors' scientific research team, this is a sort of inquiry-based learning that adopts the structure of an authentic research project. Participation in this particular project has the potential to enhance students' aptitude for critical thinking and their passion for science, as well as boost their self-assurance in conducting scientific investigations. Additionally, our undergraduate students have achieved good placements. For example, in 2022, our students applied for a project titled “Correlation Analysis of Streptococcus mutans and Oral Microorganisms in the Oral Cavity of IgA Nephropathy Patients” (Project No. S202210486281). In 2023, we successfully obtained funding for the project “Specific Detection of Porphyromonas gingivalis in vitro” (Project No. S202310486390). It is encouraging to see that some undergraduates continue their engagement in oral microbiology research even after graduation. An example is the involvement of graduate students from Hospital of Stomatology Wuhan University in conducting caries risk management based on the characteristics of caries microorganisms. This practical implementation has proven effective in helping patients with high caries risk.

Based on empirical evidence and extensive monitoring of students, our curriculum has demonstrated its worth. We are actively striving to transform the experimental course from a focus on teaching knowledge to one that emphasizes the development of practical skills. We are diligently assembling teaching practice materials and negative instances of incorrect operation, while enhancing curriculum design methodologies to elevate students' theoretical understanding, foster their innovation capacity, and grow their abilities for self-reflection and self-criticism [26–29].

### Study limitations

The study's evaluation of results based on experimental data is a valuable starting point for assessing students' progress. However, it is essential to acknowledge that students' development encompasses two stages: objective achievement and the ability to apply professional knowledge to solve practical dental problems, which requires a comprehensive skill set. Although we are actively working on developing a complete evaluation tool to assess these aspects more comprehensively, evaluating their scientific research and innovation abilities are challenging. Furthermore, due to the lack of sufficient similar studies from other institutions for comparison, gaining a deeper understanding of best practices presents a challenge.

## Conclusion

Through feedback from teachers and students, as well as comprehensive analysis of teaching outcomes, it has been demonstrated that the oral microbiology laboratory teaching enhances students' grasp of professional knowledge, improves their practical skills, strengthens their innovative abilities and overall qualities, and enhances the quality of dental education.

## Data Availability

All data generated or analyzed during this study are included in this published article.
